# 8e Protects against Acute Cerebral Ischemia by Inhibition of PI3Kγ-Mediated Superoxide Generation in Microglia

**DOI:** 10.3390/molecules23112828

**Published:** 2018-10-31

**Authors:** Linna Wang, Xiaoli Wang, Tingting Li, Yihua Zhang, Hui Ji

**Affiliations:** 1 State Key Laboratory of Natural Medicines, China Pharmaceutical University, Nanjing 210009, Jiangsu, China; aprilwln@stu.cpu.edu.cn (L.W.); lttcpu@cpu.edu.cn (T.L.); 2 Department of Pharmacology, School of Pharmacy, China Pharmaceutical University, Nanjing 210009, Jiangsu, China; 3 Key Laboratory of Carbohydrate Chemistry and Biotechnology, Ministry of Education, School of Biotechnology, Jiangnan University, Wuxi 214122, Jiangsu, China; xiaoliwang@jiangnan.edu.cn; 4 Jiangsu Key Laboratory of Drug Discovery for Metabolic Diseases, China Pharmaceutical University, Nanjing 210009, Jiangsu, China

**Keywords:** hydrogen sulfide, cerebral ischemia/reperfusion, microglial activation, NOX2, PI3Kγ

## Abstract

The inflammatory response mediated by microglia plays a critical role in the progression of ischemic stroke. Phosphoinositide 3-kinase gamma (PI3Kγ) has been implicated in multiple inflammatory and autoimmune diseases, making it a promising target for therapeutic intervention. The aim of this study was to evaluate the efficacy of **8e**, a hydrogen sulfide (H_2_S) releasing derivative of 3-*n*-butylphthalide (NBP), on brain damage and PI3Kγ signaling following cerebral ischemia injury. **8e** significantly reduced sensorimotor deficits, focal infarction, brain edema and neural apoptosis at 72 h after transient middle cerebral artery occlusion (tMCAO). The NOX2 isoform of the NADPH oxidase family is considered a major enzymatic source of superoxide. We found that the release of superoxide, together with the expression of NOX2 subunits p47^phox^, p-p47^phox^, and the upstream PI3Kγ/AKT signaling were all down-regulated by **8e**, both in the penumbral region of the rat brain and in the primary cultured microglia subjected to oxygen-glucose deprivation (OGD). With the use of siRNA and pharmacological inhibitors, we further demonstrated that **8e** regulates the formation of superoxide in activated microglia through the PI3Kγ/AKT/NOX2 signaling pathway and subsequently prevents neuronal death in neighboring neurons. Our experimental data indicate that **8e** is a potential candidate for the treatment of ischemic stroke and PI3Kγ-mediated neuroinflammation.

## 1. Introduction

As one of the major causes of death and serious long-term disability, stroke produces immense health and economic burdens globally. In 2013, 6.5 million stroke deaths were reported worldwide, 67% of which were caused by ischemic stroke [1]. Intravenous thrombolysis with tissue-type plasminogen activator (tPA) is the only approved treatment for acute ischemic stroke, but the benefit is strongly time-dependent. Although the therapeutic window of tPA safely extends to three to four and a half hours after the onset of stroke symptoms [2,3], it is not sufficient for patients to receive timely thrombolytic therapy. 

A tremendous amount of work has been done on developing neuroprotective agents, which appeared to be quite credible on animal models. However, none of these agents have been demonstrated to be effective where humans are concerned. This may be partly due to overlooking the fact that all cell types in the brain, not only neurons, respond to ischemic brain injury [4]. Homeostatic signaling among cell types maintains normal brain function, while disordered signaling contributes to the progression of injury after stroke [5]. Emerging evidence from recent studies now suggests that therapeutic approaches should target multiple cell types to protect their structural and functional integrity as well as interactions, especially in the salvageable penumbral region [5,6]. Intricate mechanisms are triggered within the penumbra following the onset of cerebral ischemia/reperfusion, and prominent inflammatory response is believed to play a critical role in exacerbating post-ischemic injury, especially neural damage [7,8]. As the major resident immune cells in the brain, microglia change from surveying to activated states when the neuronal death expands in the ischemic core and spreads to the penumbra, which affects the fate of microglia and their neighboring neurons [9,10]. Despite a poor reputation, the activation of microglia is now considered to be a protective response; however, one which the impaired central nervous system (CNS) can barely tolerate and which is therefore destructive [11,12]. From this perspective, we should search for novel treatments which can appropriately regulate the activation of microglia, to save vulnerable neurons from ischemic injury [11]. 

Activated microglia generate excessive amounts of reactive oxygen species (ROS), and ROS in turn promote microglial proliferation in a feed-forward manner [13,14]. Stimulation of NADPH oxidase activity is the predominant mechanism involved in ROS production following cerebral ischemia [15]. Although nearly all cell types in the brain express one or more NADPH oxidase (NOX) isoforms, microglia express the highest levels of NOX, especially NOX2 [16]. NOX2 is composed of membrane subunits (p22^phox^ and gp91^phox^) and cytosolic subunits (p47^phox^, p67^phox^, and p40^phox^). Phosphorylation of p47^phox^ induces its association with p22^phox^ and translocation to cell membranes, which in turn promotes interaction with p67^phox^ and the small GTPase Rac to form an active enzyme complex [17]. It has been suggested that AKT triggers the phosphorylation of p47^phox^ in leukocytes and endothelial cells [18,19]. The mechanism of p47^phox^ phosphorylation in microglial cells during the process of stroke is not yet fully understood.

Phosphoinositide 3-kinases (PI3Ks) regulate several key functions of immune cells ranging from development, proliferation to migration and cytokine production [20]. A substantial amount of data indicate that the Class IB isoform-PI3Kγ is correlated with many inflammatory and autoimmune diseases, making it a promising target for therapeutic intervention. Numerous efforts have focused on the compelling effects of phosphoinositide 3-kinase gamma (PI3Kγ) in systemic inflammation [21,22,23], but less attention is paid to its contribution to CNS inflammatory response in spite of the high abundance in the brain [24]. It has been shown that genetic and pharmacological inhibition of PI3Kγ prevented Alzheimer’s disease and surgical brain injury [25,26]. However, there is still debate about the role of PI3Kγ during stroke because although genetic deletion of PI3Kγ reduced cerebral ischemic injury in some studies [27,28], it worsened the brain damage in others [29]. Moreover, the cellular location of PI3Kγ in the CNS is also controversial [27,30]. Therefore, further investigation of the role of PI3Kγ signaling in neuroinflammation is urgently needed. 

**Figure 1:**
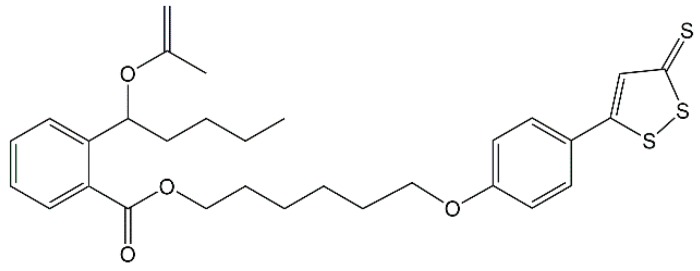
Chemical structure of **8e**.

Hydrogen sulfide (H_2_S) is well known as the third gasotransmitter beside nitric oxide (NO) and carbon monoxide (CO) [31]. Many researchers have found that exogenous H_2_S could attenuate ischemic injury in various organs and tissues, including the heart, liver and kidney [32,33,34]. The protective effects of H_2_S-releasing compounds, especially the ones with H_2_S slow-releasing donors, in cerebral ischemia is also discussed [35], but the underlying mechanism has not been thoroughly investigated. Our previous work has demonstrated that **8e** (Figure 1), an NBP-derivative combined with H_2_S donor ADT-OH (5-(4-hydroxyphenyl)-3*H*-1,2-dithiole-3-thione), exhibiting antiplatelet and antithrombotic activities, was a potential agent for the treatment of ischemic stroke [36]. Recent reports by one group showed that ADT-OH suppressed inflammatory response in microglial cells [37] and ADT conferred neuroprotection through regulating NOX4-derived ROS [38]. At the same time, several reports also showed that H_2_S donor exerts neuroprotection through anti-inflammatory effects in microglia [39,40,41] and anti-oxidative effects via NADPH oxidases [42,43,44]. We hypothesize that the essential mechanisms of H_2_S and **8e** during stroke are involved with microglial activation following oxidative stress. 

In the present study, we assessed the therapeutic effects of **8e** by studying the sensorimotor dysfunction and brain injury in a rat model of focal ischemic stroke induced by transient middle cerebral artery occlusion (tMCAO). Further investigation into the involvement of NOX2-derived superoxide and PI3Kγ signaling in the protective mechanism of **8e** was conducted. To replicate the condition of microglia and the interaction of neuron-microglia after stroke, primary microglia cultures were exposed to oxygen–glucose deprivation (OGD). **8e** down-regulated the PI3Kγ/AKT/NOX2 pathway in microglial cells and subsequently attenuated neuronal death in vitro. Our results indicate that **8e** is a promising candidate for the treatment of ischemic stroke.

## 2. Results

### 2.1. **8e** Enhanced Sensorimotor Function after Cerebral I/R in Rats

**Figure 2:**
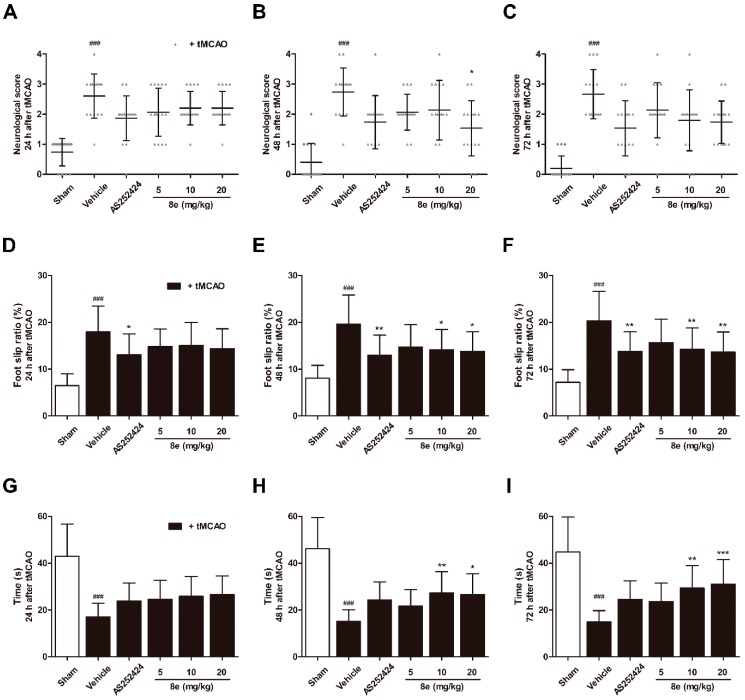
**8e** enhanced sensorimotor function after cerebral ischemia/reperfusion (I/R) in rats. (**A**–**C**) Neurological scores in Longa test; (**D**–**F**) foot slip ratios in beam walking test; (**G**–**I**) time spent on the rope in prehensile traction test at 24 h, 48 h and 72 h after transient middle cerebral artery occlusion (tMCAO). Data are presented as mean ± SD (*n* = 15). ^###^ *p* < 0.001 vs. sham group. * *p* < 0.05, ** *p* < 0.01, *** *p* < 0.001 vs. tMCAO + vehicle group.

Middle cerebral artery occlusion (MCAO) in rats induces lesions in the cortical and striatal regions which are involved in the processing of sensorimotor information. Impairment of behavior may reflect changes in brain function. A series of behavioral assessments was conducted to determine the neurological and sensorimotor deficits at 24 h, 48 h and 72 h following transient middle cerebral artery occlusion (tMCAO). In the Longa test, which offers a graded scale of body movement post ischemic injury, a repeated measures ANOVA showed a significant group effect (F = 41.88, *p* < 0.0001), but no time or interaction effect (F = 2.823, *p* = 0.061; F = 0.810, *p* = 0.620). Rats in the tMCAO + vehicle group showed severe neurological impairment at every time point tested (*p* < 0.001, respectively; Figure 2A–C). Trends of functional improvement were observed in the **8e** treatment groups, while significant differences were only found between 20 mg/kg **8e** and tMCAO + vehicle group at 48 h after tMCAO (*p* < 0.05). 

A significant group effect (F = 33.36, *p* < 0.0001) in the beam walking test, which reflects motor coordination, was observed without time or interaction effect (F = 0.282, *p* = 0.755; F = 0.374, *p* = 0.957). Post hoc analyses showed that beam walking ability was remarkably impaired in vehicle-treated animals for three days after tMCAO (*p* < 0.001, respectively; Figure 2D–F). The foot slip ratios were notably decreased in rats treated with 10 mg/kg and 20 mg/kg **8e** at 48 h (*p* < 0.05, respectively) and 72 h after tMCAO (*p* < 0.01, respectively). AS252424, a PI3Kγ pharmacological inhibitor, also reduced foot slip ratios and showed statistical differences in the beam walking test. 

The prehensile traction test primarily measures impaired forelimb placement and muscle strength by the length of time spent on the rope. A significant group effect (F = 49.25, *p* < 0.0001) but no time or interaction effect (F = 0.509, *p* = 0.602; F = 0.473, *p* = 0.907) was found. At 24 h, 48 h and 72 h after tMCAO, the vehicle-treated rats spent shorter lengths of time on the rope (*p* < 0.001, respectively; Figure 2G–I). **8e** at a dose of 20 mg/kg profoundly increased muscle strength at 48 h and 72 h after tMCAO (*p* < 0.05, *p* < 0.001, respectively). Similar results were obtained after **8e** treatment of 10 mg/kg at the same two time points (*p* < 0.01, respectively). These findings suggested that **8e** could improve behavioral outcomes after tMCAO through three days of treatment.

### 2.2. **8e** Exerted Protective Effect against Cerebral I/R Injury, Histological Damage and Neural Apoptosis in Rats

**Figure 3:**
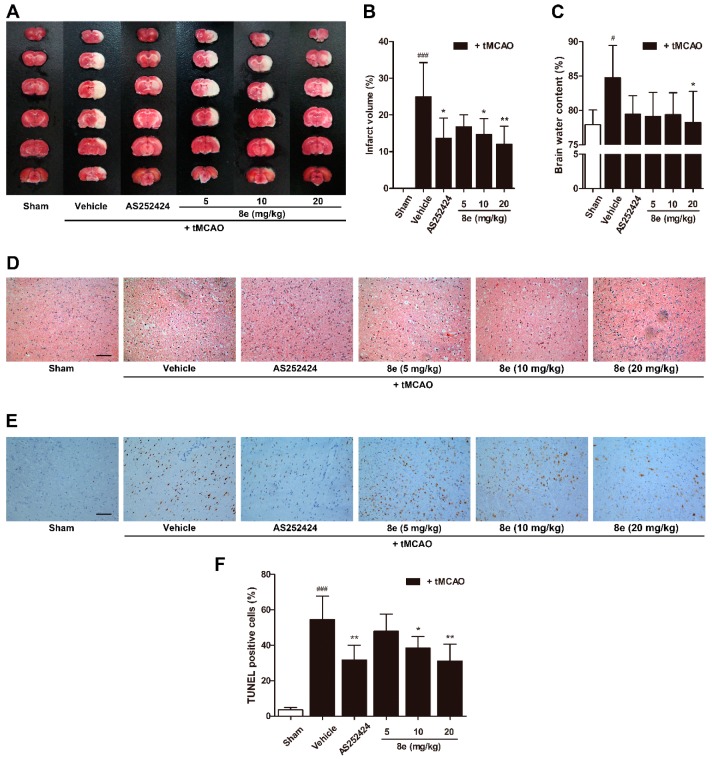
**8e** protected against cerebral I/R injury, histological damage and neural apoptosis at 72 h after tMCAO in rats. (**A**) Representative images of TTC staining on rat brain sections; (**B**) quantitative analysis of infarct volumes in TTC staining (*n* = 6); (**C**) brain water contents in ipsilateral hemispheres (*n* = 6); (**D**) representative images of H&E staining; (**E**) TUNEL staining at 200 × magnification. Scale bar = 100 μm; (**F**) quantitative analysis of the percentage ratios of TUNEL positive cells (*n* = 3). Data are presented as mean ± SD. ^#^ *p* < 0.05, ^###^ *p* < 0.001 vs. sham group. * *p* < 0.05, ** *p* < 0.01 vs. tMCAO + vehicle group.

Brain infarct volume after tMCAO was tested by TTC staining to quantify morphological changes (Figure 3A,B). The infarctions declined in rats treated with **8e** at 10 mg/kg (*p* < 0.05) and 20 mg/kg (*p* < 0.01) compared with the tMCAO + vehicle group. At 72 h after reperfusion, water contents were increased in the ipsilateral hemispheres of the vehicle group (*p* < 0.05, Figure 3C). All **8e** treatment decreased brain edema, but statistical significance was found only in the 20 mg/kg group (*p* < 0.05).

As shown in Figure 3D, tMCAO surgery induced severe histological damage in rats treated with a vehicle based on hematoxylin and eosin (H&E) staining. The presence of all three doses of **8e** notably reduced the numbers of pyknotic nuclei and vacuolization. TUNEL staining was performed to investigate whether **8e** could affect neural apoptosis following I/R (Figure 3E). The results showed that the numbers of TUNEL positive nuclei, i.e., the apoptotic nuclei, in the **8e**-treated groups were much fewer than those in the tMCAO + vehicle group (10 mg/kg, *p* < 0.05; 20 mg/kg, *p* < 0.01). Similarly, PI3Kγ-selective inhibitor AS252424 also exhibited marked protection against I/R injury, proving that inhibition of PI3Kγ is a potential approach to treat cerebral ischemia. 

### 2.3. **8e** Attenuated Superoxide Production Following Cerebral I/R in Rats

**Figure 4:**
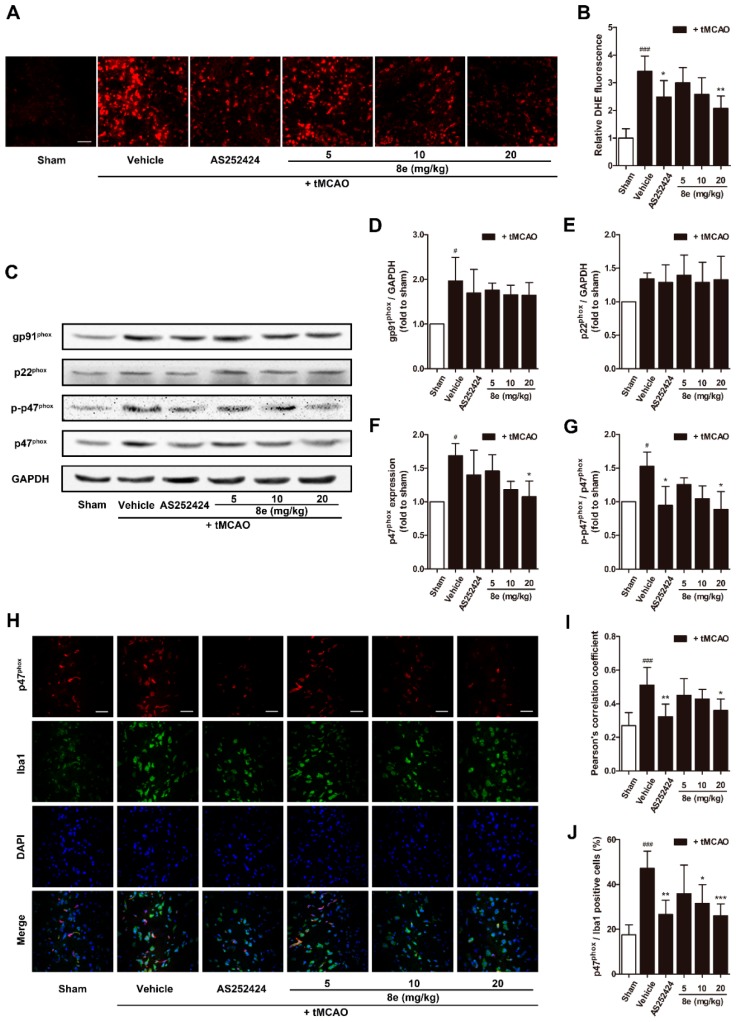
**8e** attenuated superoxide production and the expression of p47^phox^ in microglia at 72 h after tMCAO in rats. (**A**) Representative images of DHE fluorescence in penumbral region. Scale bar = 50 μm; (**B**) semiquantitative analysis of superoxide production in DHE fluorescence; (**C**) representative bands of Western blot assay for gp91^phox^, p22^phox^, p47^phox^ and p-p47^phox^ (Ser328) in penumbral region; (**D**–**G**) semiquantitative analysis of gp91^phox^, p22^phox^, p47^phox^ and p-p47^phox^ protein levels; (**H**) representative confocal fluorescence images of penumbral region labeled with p47^phox^ (red), Iba1 (green) antibodies and DAPI (blue). Scale bar = 50 μm; (**I**) Pearson’s correlation coefficients between p47^phox^ (red) and Iba1 (green) fluorescence; (**J**) quantitative analysis of the percentage ratios of p47^phox^/Iba1 positive cells to Iba1 positive cells. Data are presented as mean ± SD (*n* = 3). ^#^ *p* < 0.05, ^###^ *p* < 0.001 vs. sham group. * *p* < 0.05, ** *p* < 0.01, *** *p* < 0.001 vs. tMCAO + vehicle group.

ROS are critical mediators in the pathology of acute ischemic stroke. Superoxide is one of the most important ROS which can directly cause oxidative damage to nearby neural cells or can interact with other molecules to generate “secondary” radicals [45]. The effect of **8e** on superoxide production was evaluated with the DHE staining technique which produces red fluorescence when oxidized by superoxide and intercalates into DNA. At 72 h after the tMCAO, although limited fluorescent signal was detected in sham-operated rats, significantly higher fluorescence was found in the penumbral region of vehicle-treated ones (Figure 4A,B). Treatment with **8e** markedly attenuated the production of superoxide (20 mg/kg, *p* < 0.01) as did AS252424 (*p* < 0.05). 

We next determined the protein expression of NOX2 subunits which are regarded as the major source of superoxide in brain ischemia (Figure 4C–G). In this experiment, NOX2 was activated after tMCAO, and the levels of its subunits were significantly increased (*p* < 0.05, respectively) except p22^phox^. **8e** did not affect the elevation of gp91^phox^ although it effectively attenuated the expression of p47^phox^ and phosphorylation of p47^phox^ at Ser328 (20 mg/kg, *p* < 0.05, respectively). Meanwhile, AS252424 altered the levels of the p-p47^phox^ subunit with statistical significance (*p* < 0.05). 

As mentioned above, abundant expression of NOX2 was found in microglia [16]. We further explored whether **8e** could inhibit the activation of NOX2 in this cell type during tMCAO with double immunostaining of p47^phox^ and Iba1 antibodies. Results in Figure 4H–J showed that the Pearson’s correlation coefficient of the vehicle-treated group was sharply raised at 72 h after tMCAO (*Rr* = 0.512, *p* < 0.001). Administration of **8e** and AS252424 decreased the value of the Pearson’s correlation coefficient (20 mg/kg, *Rr* = 0.360, *p* < 0.05; AS252424, *Rr* = 0.323, *p* < 0.01). The ratios of p47^phox^/Iba1 positive cells were profoundly reduced after **8e** treatment at doses of 10 mg/kg and 20 mg/kg (*p* < 0.05, *p* < 0.001), while AS252424 had a similar effect (*p* < 0.01). These data indicated that **8e** prevents tMCAO-induced elevation of p47^phox^ level in microglial cells. We therefore presumed that the effects of **8e** on superoxide production may occur partly through inhibition of p47^phox^ and p-p47^phox^ in microglia after cerebral I/R in rats. Suppression of PI3Kγ by AS252424 could also decrease ROS generation and p47^phox^ activation, indicating potential implication of PI3Kγ signaling in the regulation of superoxide formation.

### 2.4. **8e** Inhibited PI3Kγ Signaling in Microglia after Cerebral I/R in Rats

**Figure 5:**
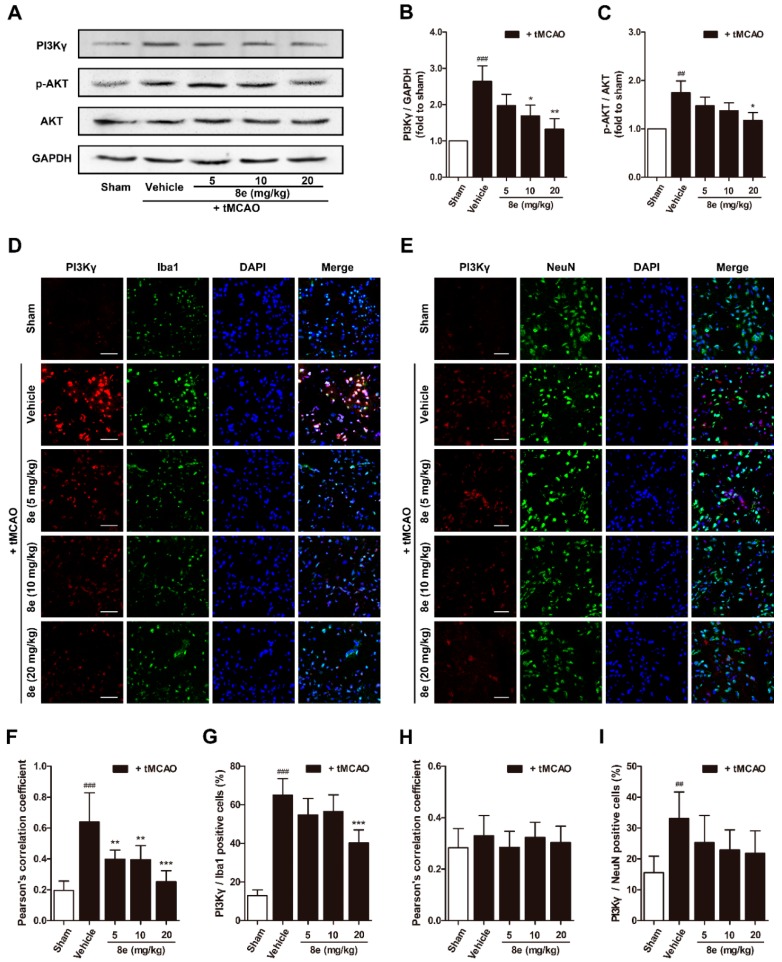
**8e** inhibited phosphoinositide 3-kinase gamma (PI3Kγ) signaling in microglia at 72 h after tMCAO in rats. (**A**) Representative bands of Western blot for PI3Kγ, AKT and p-AKT in penumbral region; (**B**,**C**) semiquantitative analysis of PI3Kγ and p-AKT protein levels; (**D**) representative confocal fluorescence images of penumbral region labeled with PI3Kγ (red), Iba1 (green) antibodies and DAPI (blue). Scale bar = 50 μm; (**E**) representative confocal fluorescence images of penumbral region labeled with PI3Kγ (red), NeuN (green) antibodies and DAPI (blue). Scale bar = 50 μm; (**F**) Pearson’s correlation coefficients between PI3Kγ (red) and Iba1 (green) fluorescence; (**G**) quantitative analysis of the percentage ratios of PI3Kγ/Iba1 positive cells to Iba1 positive cells; (**H**) Pearson’s correlation coefficients between PI3Kγ (red) and NeuN (green) fluorescence; (**I**) quantitative analysis of the percentage ratios of PI3Kγ/NeuN positive cells to NeuN positive cells. Data are presented as mean ± SD (*n* = 3). ^##^ *p* < 0.01, ^###^ *p* < 0.001 vs. sham group. * *p* < 0.05, ** *p* < 0.01, *** *p* < 0.001 vs. tMCAO + vehicle group.

Phosphorylation of AKT is a ubiquitous response to activation of Class I PI3K and has been suggested to trigger p47^phox^-mediated ROS formation in leukocytes and endothelial cells [46]. We hypothesized that **8e** may affect p47^phox^ through the PI3Kγ/AKT signaling pathway in activated microglial cells after stroke. According to our data, PI3Kγ expression in the penumbral region is notably increased at 72 h following tMCAO, and this elevation was effectively eliminated by **8e** (10 mg/kg, *p* < 0.05; 20 mg/kg, *p* < 0.01). The protein level of p-AKT was also reduced with the presence of **8e** (20 mg/kg, *p* < 0.05; Figure 5A–C). These results revealed that **8e** could inhibit the PI3Kγ/AKT pathway in the context of stroke. 

Considering the controversial location of PI3Kγ in the CNS, we further assayed the cellular distribution of PI3Kγ in the penumbral region of the ischemic brain with a double immunostaining technique (Figure 5D,E). PI3Kγ signals rose at 72 h after MCAO and were highly co-localized with Iba1 but not with NeuN, the marker for the soma of neurons. The Pearson’s correlation coefficient of the vehicle-treated group in PI3Kγ/Iba1 immunostaining exhibited a dramatic increase at 72 h after tMCAO (*Rr* = 0.638, *p* < 0.001), while this value in PI3Kγ/NeuN immunostaining was barely changed (*Rr* = 0.330, *p* > 0.05), and those of the **8e**-treated groups were reduced (5 mg/kg, *Rr* = 0.398, *p* < 0.01; 10 mg/kg, *Rr* = 0.395, *p* < 0.01; 20 mg/kg, *Rr* = 0.251, *p* < 0.001). The ratio of PI3Kγ/Iba1 positive cells was remarkably reduced when given 20 mg/kg of **8e** (*p* < 0.001). Meanwhile, the ratios of PI3Kγ/NeuN-positive cells were not observably decreased in the presence of **8e**. Our data showed that PI3Kγ was highly expressed in the microglia of the ischemic brain, and **8e** could prevent cerebral I/R injury via modulating the PI3Kγ/AKT signaling pathway in microglia. 

### 2.5. **8e** Down-Regulated PI3Kγ Signaling in OGD-Treated Primary Microglial Cells In Vitro

**Figure 6:**
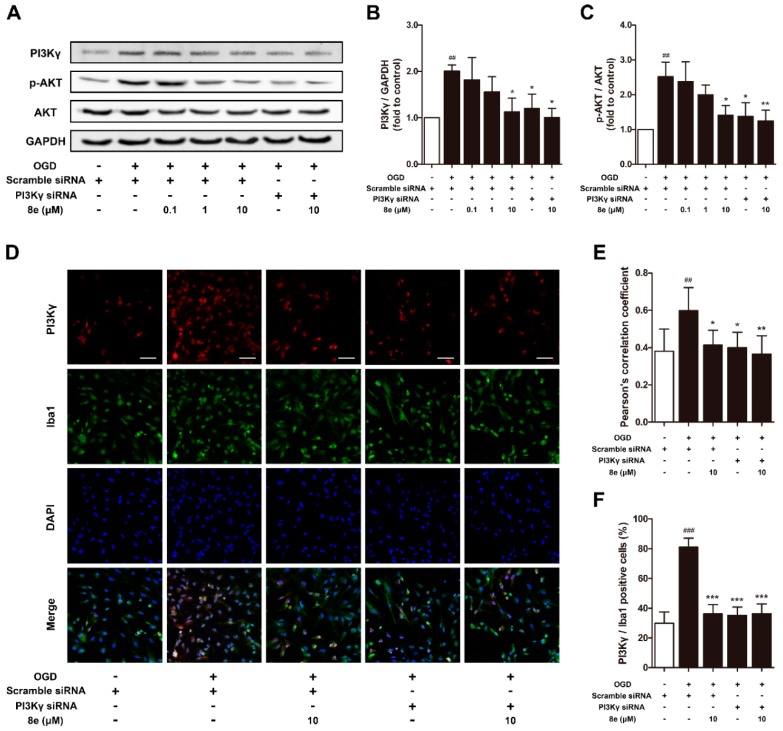
**8e** downregulated PI3Kγ signaling in primary microglial cells at 24 h after oxygen–glucose deprivation (OGD). (**A**) Representative bands of Western blot for PI3Kγ, AKT and p-AKT in primary microglia; (**B**,**C**) semiquantitative analysis of PI3Kγ and p-AKT protein levels; (**D**) representative confocal fluorescence images of primary microglia labeled with PI3Kγ (red), Iba1 (green) antibodies and DAPI (blue). Scale bar = 50 μm; (**E**) Pearson’s correlation coefficients between PI3Kγ (red) and Iba1 (green) fluorescence; (**F**) quantitative analysis of the percentage ratios of PI3Kγ/Iba1 positive cells to Iba1 positive cells. Data are presented as mean ± SD (*n* = 3). ^##^ *p* < 0.01, ^###^ *p* < 0.001 vs. control group. * *p* < 0.05, ** *p* < 0.01, *** *p* < 0.001 vs. OGD group.

We further conducted in vitro experiments to confirm whether **8e** could affect the PI3Kγ/AKT signaling pathway in primary cultured microglial cells. As expected, PI3Kγ siRNA down-regulated the levels of PI3Kγ and p-AKT in microglia subjected to OGD (*p* < 0.05, respectively; Figure 6A–C). 10 μM **8e** significantly decreased the expression of PI3Kγ and p-AKT (*p* < 0.05, respectively) and so did its combination with PI3Kγ siRNA (PI3Kγ, *p* < 0.05; p-AKT, *p* < 0.01).

In addition, for double immunostaining of PI3Kγ and Iba1 antibodies, the value of Pearson’s correlation coefficient (*Rr* = 0.400, *p* < 0.05) and the ratio of PI3Kγ/Iba1 positive cells (*p* < 0.001) were much lessened in primary microglia by the blocking of PI3Kγ with siRNA (Figure 6D–F). 10 μM **8e** and its combination with PI3Kγ siRNA exhibited similar inhibition on the fluorescent signal of PI3Kγ: Pearson’s correlation coefficient (**8e**, *Rr* = 0.413, *p* < 0.05; **8e** + PI3Kγ siRNA, *Rr* = 0.365, *p* < 0.01) and the ratios of PI3Kγ/Iba1 positive cells (*p* < 0.001, respectively) were considerably reduced. However, no significant difference was found when comparing these two groups (*p* > 0.05, respectively). These findings were consistent with our in vivo work showing that **8e** could regulate PI3Kγ signaling in microglial cells. 

### 2.6. **8e** Modulated NOX2 through PI3Kγ/AKT Signaling in Microglia Subjected to OGD In Vitro

**Figure 7:**
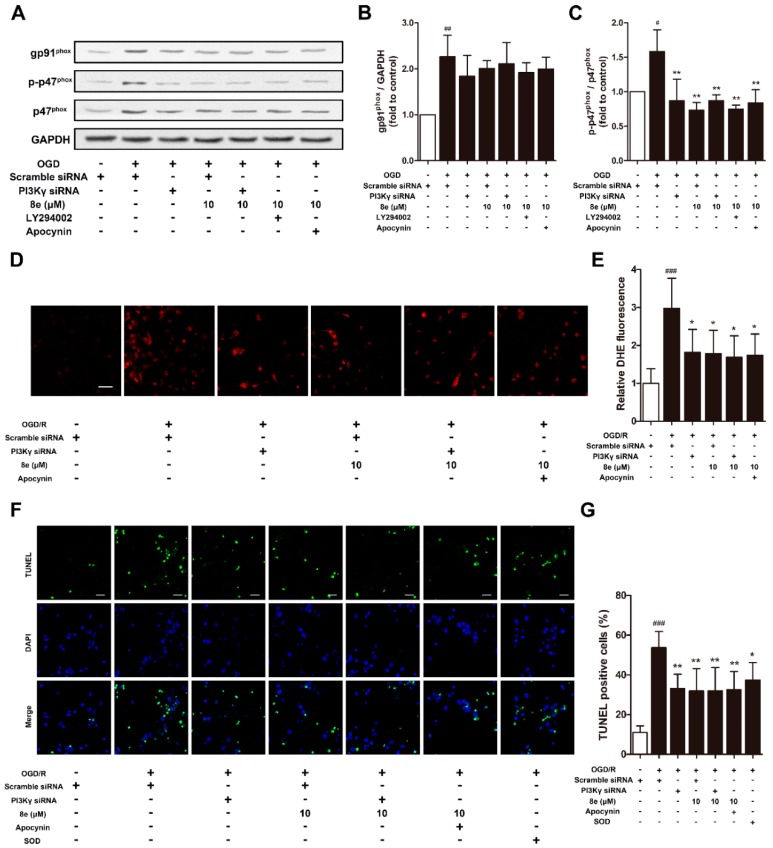
**8e** modulated NOX2 through PI3Kγ/AKT signaling in primary microglial cells and protected neurons from apoptosis at 24 h after OGD. (**A**) Representative images of Western blot for gp91^phox^, p47^phox^ and p-p47^phox^ expressions in primary microglia; (**B**,**C**) semiquantitative analysis of gp91^phox^ and p-p47^phox^ protein levels; (**D**) representative DHE fluorescence images of primary microglia at 24 h after OGD. Scale bar = 50 μm; (**E**) semiquantitative analysis of superoxide production in DHE fluorescence images; (**F**) representative TUNEL images of primary neurons counterstained with DAPI (blue). Scale bar = 20 μm; (**G**) quantitative analysis of the percentage ratios of TUNEL positive cells. Data are presented as mean ± SD (*n* = 3). ^#^ *p* < 0.05, ^##^ *p* < 0.01, ^###^ *p* < 0.001 vs. control group. * *p* < 0.05, ** *p* < 0.01, vs. OGD group.

As shown in Figure 7A–C, protein expressions of NOX2 subunits after OGD were monitored to confirm whether **8e** regulates NOX2 through PI3Kγ signaling in primary microglia. Cells were treated with LY294002 and apocynin as PI3K/AKT and NOX2 inhibitors, respectively. The level of gp91^phox^ was affected by neither 10 μM **8e** nor its combination with PI3Kγ siRNA, LY294002, or even apocynin. These results indicated that **8e** and apocynin do not modulate NOX2 via the gp91^phox^ subunit. On the other hand, the elevation of p-p47^phox^ caused by OGD was eliminated by **8e** (*p* < 0.01). Combinations of **8e** with neither PI3Kγ siRNA, LY294002 nor apocynin further decrease the expression of p-p47^phox^ when compared with **8e** (*p* > 0.05, respectively). All these data demonstrated that **8e** modulates the expression of p47^phox^ and its phosphorylation primarily through blocking of the PI3Kγ/AKT pathway in OGD-treated microglia. 

### 2.7. **8e** Protected Neurons against Apoptosis by Down-Regulating Superoxide Production in OGD-Treated Primary Microglia In Vitro

Activated microglia can induce and amplify cell damage to neighboring neurons by the excess production of cytotoxic factors such as superoxide, nitric oxide (NO) and tumor necrosis factor-α (TNFα) [7]. In this part, we investigated whether **8e** exerts neuroprotection through inhibition of superoxide formation in microglia subjected to OGD, with DHE staining. Administration of **8e** profoundly ameliorated the production of DHE fluorescence (*p* < 0.05, Figure 7D,E), while its combination with neither PI3Kγ siRNA nor apocynin further enhanced this effect in primary cultured microglia after OGD (*p* > 0.05, respectively).

Next, we found that neuronal apoptosis of TUNEL staining ascended sharply following co-culture of naïve neurons with OGD-treated microglia (*p* < 0.001, Figure 7F,G). Single administration of **8e** and PI3Kγ siRNA remarkably reduced the ratios of TUNEL positive cells (*p* < 0.01, respectively), while its combination with neither PI3Kγ siRNA nor apocynin decreased the number further (*p* > 0.05, respectively). We noticed that treatment with superoxide dismutase-1 (SOD) in culture media also attenuated neuronal apoptosis, but with less potency (*p* < 0.05). This finding partly confirmed that the superoxide generated in activated microglia enters extracellular space and spreads to nearby neurons [47]. Taken together, these results suggested that **8e** could reduce the release of superoxide by microglia, and in turn protect neighboring neurons against apoptosis.

## 3. Discussion

As a gasotransmitter, H_2_S can rapidly travel across cell membranes without specific transporters, and hence has an impact on diverse physiological and pathological progresses [48]. A growing number of studies have shown that H_2_S exerts cytoprotective activity primarily through anti-oxidative, anti-inflammatory, anti-apoptotic and pro-angiogenesis actions [37,49,50,51]. However, H_2_S gas is not an ideal resource due to difficulties in concentration control and the risk of excessive toxicity [52]. Inorganic sulfide salts such as sodium sulfide (Na_2_S) and sodium hydrogen sulfide (NaHS) also release H_2_S rapidly and spontaneously. Recently, a series of slow-releasing H_2_S donors and hybrid drugs have been developed as new therapeutic approaches. They generate H_2_S with lower levels of release rates and longer treatment intervals, in contrast to inorganic sulfide salts. A study by Whiteman et al. indicated that the effects of H_2_S on inflammation depend not only on H_2_S concentration but also on the rate of H_2_S generation [53]. That may be one explanation for the seemingly controversial role of H_2_S in inflammation-related diseases, such as stroke [38,54]. ADT and its main metabolite ADT-OH have been used extensively as a slow-releasing donor of H_2_S [55]. Combinations of ADT-OH with L-DOPA and aspirin have shown protective properties in neurodegenerative processes [56,57]. 

3-*n*-butylphthalide (NBP) was approved by the China Food and Drug Administration (CFDA) in 2002 as a new drug for ischemic stroke treatment. Our group investigated a series of NBP derivatives combined with ADT-OH and reported that oral treatment with **8e** provides superior antiplatelet and antithrombotic activities than NBP [36]. In this study, we verified the therapeutic effect of **8e** in a rat model of transient focal cerebral ischemia. After a three-day intravenous treatment post tMCAO, **8e** improved sensorimotor outcomes in three different behavioral assessments. The Longa test is one of the most frequently used neurological exams in rodent stroke studies. Although improvement trends were observed in all doses of **8e**-treated groups, statistical significance was only found at 20 mg/kg following 48 h of reperfusion. This may be partly due to the fact that the Longa test focuses only on motor deficits. A recent study recommended the use of both Garcia and Longa tests to capture a broader range of neurological deficits [58]. The beam walking and prehensile traction tests are also widely applied in models of brain damage [59]. **8e** markedly reduced the foot slip ratios and increased the length of time spent on the rope at 48 h and 72 h after tMCAO, indicating a protective capability of **8e** in the sub-acute phase of ischemic stroke (Figure 2D–I). Meanwhile, we found that infarction volume and brain edema were notably decreased in the presence of **8e**, which provided solid evidence for its effectiveness. The results of histological examination and TUNEL staining in the penumbral region further confirmed these findings (Figure 3). 

After stroke, inflammatory response is initiated in ischemic penumbra which is a metabolically active, but neurophysiologically silent, region surrounding the infarct core [60]. This response is characterized by rapid activation of microglia, production of proinflammatory mediators, and infiltration of various types of inflammatory cells including neutrophils, monocytes/macrophages, T cells and other cells [61]. Resident microglial cells are among the first cells to respond to brain injury. They are activated within minutes of ischemia onset and peak at 48–72 h [62]. Considering that activated microglia are morphologically and functionally indistinguishable from blood-derived monocytes/macrophages in the brain, Schilling et al. designed a series of experiments and confirmed that blood-derived macrophages are recruited at a later time and become abundant on days 3–7 [63,64,65]. Resident microglial activation precedes and predominates over blood-derived macrophage infiltration during the first three or four days of cerebral ischemia. Invasion of neutrophils occurs slightly later at 48 h. However, the existence of neutrophils is largely masked after day three by the large-scale accumulation of activated microglia and macrophages in the penumbra region [66]. 

The experimental observations that PI3Kγ is an important mediator in the signaling cascade of inflammatory response, have attracted worldwide attention to this kinase. But the role of PI3Kγ in the context of brain injury, has not been extensively studied. The level of PI3Kγ was significantly increased at one day and, up to seven days, after surgical brain injury in rats [26]. Similar up-regulation was found by Jin et al. in a mouse model of tMCAO within 24 h [27]. Here we reported for the first time that the protein expression of PI3Kγ is elevated in the penumbral region of rats at 72 h following cerebral ischemia (Figure 5A,B). We also detected the cellular location of PI3Kγ by a double immunostaining technique. PI3Kγ was colocalized with Iba1, a cell marker for microglia, while limited fluorescence signals of PI3Kγ were expressed in NeuN-positive neurons (Figure 5D–I). These data indicated that microglia may be the major cellular source of PI3Kγ in the penumbral region at day three after ischemic stroke. Our findings are consistent with the time course of inflammatory cell recruitment motioned above, suggesting PI3Kγ’s involvement in microglial activation after tMCAO. Genetic ablation and pharmacological inhibition of PI3Kγ confer enhanced protection against Alzheimer’s disease, surgical brain injury and cerebral ischemia [25,26,28]. On the other hand, the selective PI3Kγ inhibitor AS252424 at a dose of 10 mg/kg significantly attenuated surgical brain injury-induced brain edema and neurological deficits at 24 and 72 h [26]. In our research, administration of AS252424, a PI3Kγ-selective inhibitor, attenuated sensorimotor deficits, infarction size and neural apoptosis following tMCAO (Figure 2 and Figure 3), proving that inhibition of PI3Kγ is a potential therapeutic strategy for neuroinflammation-mediated disorders. At the same time, **8e** effectively down-regulated the level of PI3Kγ and improved I/R injury in vivo and in vitro (Figure 5 and Figure 6). The combination of **8e** with PI3Kγ siRNA did not further reduce the protein expression in primary cultured microglia, indicating that **8e** exerts protective effect primarily through inhibition of PI3Kγ signaling. Phosphatase and tensin homologue deleted on chromosome 10 (PTEN) negatively regulates the PI3K pathway and AKT activation. Recent reports showed that treatment with 25–100 μM NaHS reduced PTEN expression, whereas 800–1000 μM NaHS increased the level of PTEN protein in hepatocellular carcinoma cells [67]; however, the relative roles of H_2_S donor in PTEN regulation in innate immune cells are still mostly unexplored.

Oxidative stress is implicated in acute brain injury and chronic neurodegenerative disorders [68]. Because of its high metabolic rate and relatively reduced capacity for cellular regeneration, the brain is extremely vulnerable to oxidative damage [69]. Unfortunately, therapies based on free radical scavenger or antioxidant principles have generally failed in clinical trials, despite the promising efficacy in preclinical studies. More and more researchers have shifted their focus onto ROS-generating pathways in various pathological situations. NADPH oxidases are considered the major source of ROS production, among which NOX2 is one of the most extensively studied isoforms during ischemic injury [70,71]. A strong increase of NOX2 was reported in resident microglial cells and recruited neutrophils after stroke onset [72]. A previous study has detected the robust production of superoxide at 4 h and its decrease within 24 h after reperfusion in WT mice, which were attributed to the enhanced neutrophil infiltration into the ischemic area [28]. They also found that the protein expression of NOX2 continuously increased until 72 h after tMCAO in WT mice. In the present study, elevation in the expression of NOX2 subunits and superoxide were observed at 72 h following cerebral ischemia in rats. This ROS generation and NOX2 activation at later time-points appears to be the consequence of microglial response to brain injury, as the infiltration of neutrophils has not reached its peak and their existence is masked in the sub-acute phase of stroke. Inhibition of PI3Kγ with AS252424 showed significant reductions in superoxide formation, the protein level of p-p47^phox^ and the fluorescence signal of p47^phox^ in microglia at 72 h after tMCAO (Figure 4). Similar results were found with the use of PI3Kγ siRNA in primary cultured microglia following OGD. **8e** also exhibited equivalent effects on regulation of phosphorylated p47^phox^ and NOX2-derived superoxide in vivo and in vitro (Figure 7A–E). Furthermore, combination of **8e** with neither PI3Kγ siRNA nor apocynin enhanced this effect in microglial cells. On the other hand, AKT is an established downstream of Class I PI3K, and it has been suggested that it is involved in the activation of p47^phox^ in leukocytes and endothelial cells [18,19]. Our experimental data showed that, both **8e** and blocking PI3Kγ eliminated the increase of p-AKT following I/R injury. The combination of **8e** with LY294002, a PI3K/AKT inhibitor, did not further decrease the expression of p-p47^phox^ when compared with **8e**. Taken together, these results demonstrate that **8e** modulates p47^phox^ and its phosphorylation through PI3Kγ/AKT signaling. With the use of a co-culture model, we also observed that superoxide generation from activated microglia enters extracellular space and spreads to nearby neurons. Administration of **8e** and PI3Kγ siRNA to OGD-treated microglia markedly attenuated neuronal apoptosis in naïve neurons, suggesting that **8e** protects neighboring neurons from apoptosis via control of superoxide generation in microglia (Figure 7F,G). 

## 4. Materials and Methods 

### 4.1. Chemicals

**8e** (purity >98%) was synthesized by Jiangsu Key Laboratory of Drug Discovery for Metabolic Diseases, China Pharmaceutical University. PI3Kγ-selective inhibitor AS252424, PI3K/AKT inhibitor LY294002, NOX2 inhibitor apocynin and superoxide dismutase-1 (SOD) were purchased from Aladdin Biotechnology, China. 0.9% NaCl solution containing 5% ethanol and 5% polyethylene glycol 400 (*v*/*v*, both from Sinopharm Chemical Reagent, Shanghai, China) was utilized as the vehicle. 

### 4.2. Animals

All animal experimental protocols were approved by Ethics Committee of China Pharmaceutical University and performed in compliance with the Guideline on Administration of Laboratory Animals (China). Efforts were made to minimize the number of animals used and their suffering. Adult male Sprague-Dawley rats (250–280 g) were provided by the Comparative Medicine Centre of Yangzhou University (China) and housed under standard laboratory conditions of controlled temperature, humidity and a 12-hour light/dark cycle. The animals were allowed free access to food and water during one week of adaptation and fasted overnight before surgery. 

### 4.3. tMCAO Surgery and Drug Treatment

Transient middle cerebral artery occlusion was accomplished as previously described [73] to mimic human cerebral ischemia/reperfusion (I/R) injury. Briefly, after achieving general anesthesia by intraperitoneal injection of chloral hydrate (300 mg/kg, Sinopharm Chemical Reagent, Shanghai, China), the regional cerebral blood flow (rCBF) was monitored by Iaser Doppler flowmetry (LDF 100C, BIOPAC Systems, Goleta, California, USA). A 4-0 nylon monofilament suture with a rounded tip (Sunbio Biotechnology, Beijing, China) was inserted into the right internal carotid artery (ICA) through the external carotid artery (ECA) stump and gently advanced to block the origin of the middle cerebral artery (MCA). Restoration of MCA blood flow was accomplished by withdrawing the suture 60 min later. The sham-operated rats received the same surgery without suture insertion. Rats with unsuccessful occlusion (reduction of rCBF <75%) and unsuccessful reperfusion (recovery of rCBF <70%) were excluded from the study [74]. The core body temperature was maintained at 37 ± 0.5 °C throughout the procedure. 

Before surgery, rats were randomly divided into six groups, including sham, tMCAO + vehicle, tMCAO + 10 mg/kg AS252424, tMCAO + 5 mg/kg **8e**, tMCAO + 10 mg/kg **8e**, and tMCAO + 20 mg/kg **8e** groups. Among the 120 rats that underwent the tMCAO surgery, 4 rats were excluded for unsuccessful occlusion and 5 rats were excluded for unsuccessful reperfusion; 7 rats died during the surgery and 9 after the surgery. AS252424 and other drugs were administered intraperitoneally or intravenously at the beginning of reperfusion and repeated every 24 h for 3 days. The doses of **8e** and AS252424 were calculated based on the clinical use of NBP and sodium chloride injection and previous report [26]. 

### 4.4. Behavioral Assessment

#### 4.4.1. Longa Test

This neurological assessment is a 5-point scale: 0, no neurological deficit; 1, failure to extend the left forepaw fully; 2, circling to the left; 3, falling to the left; and 4, no spontaneous walking and being depressed [75]. At 24 h, 48 h and 72 h after tMCAO, the performance of rats was evaluated by a blinded investigator. 

#### 4.4.2. Beam Walking

Fine motor coordination was assessed with the beam walking task. For 2 days before surgery, the rats were trained to traverse an elevated narrow beam (2.5 cm in width, 1.2 m in length and 0.8 m in height) to enter a darkened goal box at the opposite end of beam. The test was conducted at 24 h, 48 h and 72 h after tMCAO and the performance of the rats was videotaped and analyzed by calculating the foot slip ratios of the impaired limbs (number of foot faults/number of total steps) by a blinded investigator. A fault was defined as any foot slip off the top surface of the beam or any limb use on the side of the beam [76].

#### 4.4.3. Prehensile Traction

A 0.6 m long and 4 mm diameter wire was placed horizontally 0.4 m above a foam pad [59]. At 24 h, 48 h and 72 h after tMCAO, the animals were released with their forepaws placed onto the rope to evaluate muscle strength and equilibrium. The time to falling (maximum 60 s) was noted.

### 4.5. Infarct Volume and Brain Edema Measurement

At 72 h after tMCAO, rats were deeply anesthetized with a lethal dose of chloral hydrate (350 mg/kg) and the brains were frozen at −20 °C for 20 min. The frozen brains were cut into 2-mm-thick coronal sections and stained with 2% of 2,3,5-Triphenyltetrazolium chloride (TTC, Sigma-Aldrich, St. Louis, MO, USA) for 20 min at 37 °C. The brain slices were then fixed in 4% formaldehyde overnight at room temperature and photographed with a digital camera. The area of infarction in each slice was measured using Image J (NIH, Bethesda, MD, USA), and infarct volume was obtained from the product of average thickness (2 mm) and the sum of infarction areas in all slices examined. Infarct volume (%) = total infarct volume/total brain volume × 100%. 

Brain edema was evaluated by measuring the brain water content as previously described [77]. Brains were rapidly removed at 72 h post reperfusion and both hemispheres were weighed before and after drying at 100 °C for 24 h. The brain water content (%) = (1—dry weight/wet weight) × 100%. 

### 4.6. Primary Microglia Culture 

Microglial culture was prepared as previously described with modification [74]. The brain of postnatal (P0-2) Sprague-Dawley (SD) rats was separated and the meninges were carefully removed. The cerebral cortex was cut into small fragments and digested with trypsin (0.25%, Sigma-Aldrich, St. Louis, MO, USA) for 10 min at room temperature. The cell suspension was filtered through 100-μm and 40-μm cell strainers (BD Falcon, Bedford, MA, USA). After centrifugation for 5 min at 100 *g*, the pallet was resuspended in Dulbecco’s modified Eagle’s medium/Ham′s nutrient mixture F-12 (DMEM/F12) containing 10% fetal bovine serum (FBS) and seeded into poly-lysine (0.1 mg/mL, Sigma-Aldrich, St. Louis, MO, USA) pre-coated 75-cm^2^ flasks in a humidified incubator (95% O_2_, 5% CO_2_, 37 °C). Culture media were changed every 2 days. Two weeks later, the isolation of microglia was accomplished by gentle shaking of the culture flask. The collected cells were seeded into 24-well plates with coverslips, or the upper inserts of Transwell chambers (0.4 μm diameter pores, Corning, NY, USA). 

### 4.7. siRNA Transfection and OGD Treatment

PI3Kγ siRNA and negative control (40 nM, all from GenePharma, Shanghai, China) were transfected into primary cultured microglia using Lipofectamine 2000 (Thermo Fisher Scientific, San Jose, CA, USA) 24 h post cell seeding. PI3Kγ siRNA consisted of an RNA duplex containing a sense strand 5′-GCA UUG AUA UCC CUG UCU UTT-3′ and an antisense strand 5’-AAG ACA GGG AUA UCA AUG CTT-3′. 

48 h after transfection, microglia were subjected to OGD by replacing the culture media with glucose free DMEM containing 2% FBS and transferring to an OGD incubator chamber (95% N_2_, 5% CO_2_, 37 °C). After 1 h of OGD, the plates were removed from the chamber and the culture media were replaced with DMEM/F12 and 2% FBS to achieve reoxygenation. Microglial cells were harvested for further experimentation 24 h post reoxygenation. Drug treatments including LY294002 (10 μM), apocynin (1 mM) and SOD (200 U/mL) were applied to the cell culture 2 h before and during OGD/reoxygenation. 

### 4.8. Primary Neuron Culture and Neuron-Microglia Co-Culture

Primary neuron cultures were prepared using embryos (E16-18) of SD rats. The cortex of the brainwas dissected and digested with 800 U DNase I and 2 mg/mL papain (both from Biosharp, Hefei, Anhui, China) at 37 °C for 30 min [78]. The cell suspension was filtered through 40-μm cell strainers (BD Falcon, Bedford, MA, USA). After centrifugation for 5 min at 100 *g*, the cells were resuspended in DMEM/F12 containing 10% FBS and seeded into poly-l-lysine pre-coated lower wells of Transwell chambers with coverslips. The culture media were replaced by Neurobasal medium supplemented with 2% B27 (both from Gibco, Carlsbad, CA, USA) after 4 h of incubation. Half of the media were replaced with fresh Neurobasal/B27 every 4 days. 

After 7–10 days of incubation, the lower wells containing naïve neurons were allowed to communicate with microglia subjected to 1 h of OGD in the upper inserts of the Transwell chambers for 24 h (95% N_2_, 5% CO_2_, 37 °C). The culture media were all replaced with Neurobasal/B27. 

### 4.9. Histological Observation and Apoptosis Analysis

Rats were anesthetized with 350 mg/kg of chloral hydrate and intracardially perfused with iced normal saline followed by 4% paraformaldehyde (PFA, Sinopharm Chemical Reagent, Shanghai, China) at 72 h after tMCAO. The brains were post-fixed in PFA overnight and embedded in paraffin. Microglia-bearing coverslips were removed from the 24-well plates 24 h after OGD, then fixed with 4% PFA for 30 min and permeabilized with 0.1% Triton X-100 (Sigma-Aldrich, St. Louis, MO, USA). For histological observation, 5-μm brain slices at the level of bregma +0.7 to −4.3 mm were counterstained with hematoxylin and eosin. Terminal deoxynucleotidyl transferase dUTP nick end labeling (TUNEL) staining were conducted on both brain slices and coverslips according to manufacturer’s instructions (KeyGEN Biotech, Nanjing, China). Tissue sections of the penumbral regions were observed under a microscope with 200× magnification (CKX41, Olympus, Tokyo, Japan). Cell TUNEL staining was captured with a confocal microscope (LSM700, Zeiss, Oberkochen, Germany). The numbers of TUNEL-positive cells were analyzed by a blinded investigator with Image J. 

### 4.10. Oxidative Stress Evaluation

72 h after tMCAO, the animals were anesthetized with 350 mg/kg of chloral hydrate and perfused with iced normal saline through the heart. 5-μm frozen brain slices at the level of bregma +0.7 to −4.3 mm were cut on a CM1950 cryostat (Leica, Wetzlar, Germany) and further fixed in acetone for 10 min at 4 °C. Microglia-bearing coverslips were prepared as mentioned above. The slides and coverslips were incubated with 2.5 μM dihydroethidium (DHE, Beyotime Biotechnology, Shanghai, China) at 37 °C for 30 min in a humidified chamber which was shielded from light [79]. DHE produces red fluorescence when oxidized to ethidium by superoxide anion and intercalates into DNA. Fluorescent images were acquired using a confocal microscope. The mean fluorescence intensity for each region (integrated density/area) of the penumbral regions was blind-analyzed by Image J and normalized to the level of the sham or control group. 

### 4.11. Immunofluorescent Staining

Frozen brain slices and microglia-bearing coverslips mentioned above were immunostained as previously described [80]. Briefly, after incubation with primary antibodies of p47^phox^ (1:200, Sigma-Aldrich, St. Louis, MO, USA), PI3Kγ (1:500, Novus, Littleton, CO, USA), Iba1 (1:500, Millipore, Billerica, MA, USA) and NeuN (1:500, Millipore, Billerica, MA, USA), antibody binding was visualized with Alexa Fluor 488-labeled goat anti-mouse IgG and Cy3-labeled goat anti-rabbit IgG (1:200, both from Beyotime Biotechnology, Shanghai, China). Cell nuclei were counterstained with 4’,6-diamidino-2-phenylindole (DAPI). Images of the penumbral regions were obtained using a confocal microscope. The Pearson’s correlation coefficient and the numbers of antibody-positive cells were analyzed by a blinded investigator using Image J. 

### 4.12. Western Blotting

Total protein was prepared from the penumbral regions at the level of bregma +0.7 to −4.3 mm 72 h after tMCAO, and primary cultured cells 24 h after OGD as reported previously [81]. Samples were homogenized in iced radio immunoprecipitation assay (RIPA) buffer supplemented with protease and phosphatase inhibitor cocktail (all from Beyotime Biotechnology, Shanghai, China). Following centrifugation at 13,000 *g* for 20 min at 4 °C, the supernatant was collected. Equal amounts of protein (20 μg) were loaded onto 8–10% sodium dodecyl sulfate polyacrylamide gel electrophoresis (SDS-PAGE) gels and transferred to polyvinylidene fluoride (PVDF) membranes (Millipore, Billerica, MA, USA). Membranes were blocked with 5% non-fat milk in Tris-Buffered Saline with Tween 20 (0.1%, *v*/*v*, TBST) for 1 h at room temperature, and incubated overnight at 4 °C with primary antibodies against PI3Kγ, gp91^phox^, p22^phox^, p47^phox^ (all from Santa Cruz Biotechnology, Dallas, TX, USA), phosphorylation-p47^phox^ at Ser328 (Abcam, Cambridge, MA, USA), AKT, phosphorylation-AKT at Ser473 and GAPDH (all from Cell Signaling Technology, Danvers, MA, USA). Then the membranes were hybridized with the appropriate horseradish peroxidase (HRP) conjugated secondary antibodies (1:5000, Cell Signaling Technology, Danvers, MA, USA) for 1 h at room temperature. Protein expression was detected by the enhanced chemiluminescence (ECL) method on an imaging system (ChmiScope 2850; Clinx Science Instruments, Shanghai, China) and normalized to internal reference. 

### 4.13. Statistical Analysis

Data are presented as the mean ± SD. All statistical analyses were performed using GraphPad Prism 5.0 (GraphPad Software, San Diego, CA, USA). All behavioral data were analyzed using repeated measures ANOVA. Group differences were ascertained using Dunn or Tukey post hoc comparisons where appropriate. As the normality test with the Kolmogorov-Smirnov test was passed, the rest of the data were analyzed by one-way ANOVA with Tukey’s post hoc test. *p* < 0.05 was considered statistically significant.

## 5. Conclusions

Our study demonstrated that **8e** attenuates sensorimotor deficits, focal infarction and neural apoptosis in cerebral ischemia. **8e** may exert protective effects partly thorough regulation of superoxide production in activated microglia via the PI3Kγ/AKT/NOX2 signaling pathway. These findings about **8e** widen our knowledge of H_2_S-releasing compounds and provide a novel direction for stroke research.
